# Plant Taxonomy, Systematics and Phylogeography

**DOI:** 10.3390/plants13192670

**Published:** 2024-09-24

**Authors:** Jacek Urbaniak, Paweł Kwiatkowski

**Affiliations:** 1 Department of Botany and Plant Ecology, Wrocław University of Environmental and Life Sciences, 50-363 Wrocław, Poland; 2 Institute of Biology, Biotechnology and Environmental Protection, University of Silesia in Katowice, 40-032 Katowice, Poland; pawel.kwiatkowski@us.edu.pl

**Keywords:** taxonomy, phylogeography, biogeography, plants, population biology, hybridization

## Abstract

Plant taxonomy and phylogeny deal with very important problems related to the genetic diversity of populations found in different geographical regions, trying to present the variability of these populations but also to explain their origin. For this reason, such studies examining the conditioned diversity of plant populations were the subject of this Special Issue (SI) of the journal Plants. This SI contains ten original articles and one review article concerning phylogeography and related sciences, i.e., biogeography, taxonomy, and systematics, as well as the genetic variability that lies at the basis of these sciences. The articles included in the SI cover a wide range of topics, including the phylogeny and taxonomy of mosses, taxonomy and hybridization and phylogeny of the genus Chery, genetic diversity and phylogeography of fern, including relict species from the Macronesia area, taxonomy of the genus Aconitum, a species of orchid new to the Romanian flora, but also a proposal for a new phylogeographic division in Gansu Province (China) and a review of the role of the Hercynian Mountains in Shaping Plant Migration Patterns in the Pleistocene.

## 1. Introduction

The aim of this Special Issue, entitled “Plant Taxonomy, Systematics and Phylogeography”, was to draw readers’ attention to current knowledge and present the most up-to-date results on issues related to phylogeography, plant taxonomy, and systematics. These issues complement each other, but the purpose of taxonomy and systematics is to study and classify the variability of taxa, while phylogeography and biogeography deal with the geographical distribution of organisms and the study of historical processes that may be responsible for the current distribution of the phylogenetic lines of populations. This Special Issue of “Plant Taxonomy, Systematics, and Phylogeography” contains eleven scientific articles (ten original scientific articles and one original review paper), which, in our opinion, contribute to a better understanding of interesting issues such as the taxonomy and phylogeography of organisms. Below is an overview of the articles included in the Special Issue. In recent years, population genetics, together with widely used various molecular studies and the resulting analyses, have been providing completely new data, strengthening existing views, or sometimes completely changing the current understanding of systematics, taxonomy, and phylogeography.

## 2. Overview

One of the published articles is an article about mosses from the Dicranaceae family in China, or more precisely, representatives of the very diverse and widespread genus *Dicranum* Hedw. However, despite this, the species diversity and distribution of this genus, not only in China but also in Europe, America, and Asia, is not well known. Therefore, Ref. [1] performed a taxonomic revision of the genus *Dicranum* in China using various research methods, both morphological and molecular. As a result, the authors confirmed the occurrence of 39 species of the *Dicranum* genus in China and also isolated new species in the article: *D. bardunovii* Tubanova & Ignatova, *D. dispersum* Engelmark, *D. schljakovii* Ignatova & Tubanova, and *D. spadiceum* J.E.Zetterst. Dicranum psathyrum Klazenga was transferred to Dicranoloma (Renauld) Renauld as a new synonym of Dicranoloma fragile Broth. Two other species, *D. brevifolium* (Lindb.) Lindb. and *D. viride* (Sull. and Lesq.) Lindb., are excluded from the bryoflora of China. In addition to the presented research results, the authors also published a key for identifying species of the *Dicranum* genus occurring in China, drawing attention to the large gaps in data regarding distribution studies.

Research paper [2] also concerned the Bryophyta phylum, specifically *Aloina catillum*, a species of very variable moss occurring in xerophytic environments and characterized by a diversified structure of the leaf margin compared to other closely related species of the *Aloina* genus. However, morphological variability is often not a sufficient solution to problems related to inter- and intraspecific variability and taxonomic relationships with related species. The published results, including analyses of molecular marker sequences and morphometric studies, suggest that *A. catillum* is a collective species composed of the species *A. bracteata* sp. nov. and *A. limbata* sp. nov.

The results of bryophyte research are also presented in the work on *Lophozia pallida*, a little-known species occurring in China and the Himalayas, belonging to the equally poorly known genus *Lophozia*, numbering from 20 to 22 species [3]. The small differences between species mean that many taxa are incorrectly interpreted, as the authors of the publication point out. Comparative studies of herbarium materials conducted and published in the SI allowed for a different look at the distribution of the entire genus and a revision of the distribution of the genus *Lophozia*. The authors also demonstrated the close genetic relationship of *L. guttulata* with the Japanese–Korean species *L. koreana*.

The next two papers published in the SI concern ferns. The first one concerns the endangered tree fern *Culcita macrocarpa*, which is an Iberian–Macaronesian endemic and the only European species of the order Cyatheales [4]. Like many species in Macronesia, it is considered a tertiary relict of the European paleotropical flora. The article explains the history and phylogeography of *C. macrocarpa*. Studies using eight newly identified microsatellite DNA loci and one plastid DNA region for 17 populations covering the species’ range indicate the phenomenon of clonality, which is quite common in *C. macrocarpa* and affects its diversity and genetic structure. Based on the results obtained, the authors identified two main population groups: one occurring in the northern part of the Iberian Peninsula and on the Macronesian islands, and the other in the south of the Iberian Peninsula with recently established relict populations. The authors believe that the current populations from the south of Spain originate, among others, from the Azores and Cantabrian Cornices and confirm that *C. macrocarpa* survived through the Last Glacial Maximum in two refuges in these regions.

The next article concerns ferns from the Marattiaceae family, which is a phylogenetically isolated family of tropical ferns that includes over one hundred species. Publication [5] concerns the phylogenetic relationships between these species, using data from 26 transcriptomes. Using phylogenetic analyses, the phylogeny of Marattiaceae was reconstructed, and data analysis allowed the identification of five genera of Marattiaceae as monophyletic genera with good statistical support. The presented results will certainly improve our knowledge of the use of the phylotranscriptomic method to study phylogeny and hybridization events for difficult taxa in ferns, not only those belonging to the Marattiaceae family.

In article [6], the authors describe a newly identified species from the Orchidaceae family: *Gymnadenia winkeliana*. Two populations of *G. winkeliana* were discovered in the Bucegi Mountains (Carpathians), and morphological analyses of individuals belonging to both populations were performed, paying attention to features of taxonomic importance, analyzing a total of 223 morphological features. The publication also presents the distribution of the taxon and its limited occurrence in high mountain areas in the central part of the Bucegi Natural Park, along with information on the habitat, ecology, phenology, protection, and distribution.

Classification of the genus *Aconitum* was the subject of paper [7], and its authors carried out their research using morphological observations and ISSR molecular markers to distinguish *A. flavum* from *A. pendulum*. This allowed for insight into the interspecific classification of the *Aconitum* genus. The results of the UPGMA, PCoA, and Bayesian structural analyses allowed for the separation of two population groups and demonstrated that both *A. flavum* and *A. pendulum* are separate species. Moreover, the authors point to a high level of genetic diversity in both species, which allows the conclusion that the markers used are efficient in distinguishing both species and are suitable for research on genetic diversity and population structure within the *Aconitum* genus.

Article [8] concerns the subgenus *Cerasus* (Rosaceae), an economically important subgenus with a high genetic diversity but still undetermined origin. For resolving the remaining issues in the origin of the genus, the authors used genetic analyses. The use of advanced analyses made it possible to estimate genetic diversity and suggest the independent evolution of the *Cerasus* and *Microcerasus* genera. The existence of two separate centers of geographical origin in Europe and China with significant genetic diversity of cherry populations from these regions was also confirmed. This is probably the result of long-term geographical isolation. Moreover, the results suggest the repeated occurrence of hybridization phenomena in glacial refuges and rapid dispersion during the Pleistocene period.

The next article concerns *Cerasus pseudocerasus* (Lindl.) G.Don and auto- or allo-polyploid origin and hybridization. The authors of [9] present the results of a genomic in situ hybridization (GISH) analysis to reveal the genomic relationships between Chinese cherry and other diploid relatives in the genus Cerasus. On this basis, the authors concluded that the Chinese cherry—*C. pseudocerasus*—is relatively closely related to *C. campanulata* and *C. conradinae*, while being distantly related to another fruiting cherry, *C. avium*. Moreover, the obtained results suggest that *C. pseudocerasus* probably originates from autotetraploidy.

Article [10] examines biogeographic diversity in Gansu, a region of significant biogeographic diversity located in northwestern China. The study results indicate that the southeastern region of Gansu showed the lowest level of spatial diversity in both phylogenetic relationships and taxonomic composition of floral communities in the entire Gansu region. Furthermore, the results showed a consistent pattern of distribution of phylogenetic lineages in the region and allowed us to identify nine distinct floristic regions in Gansu, which in turn were divided into two broader geographic units: the northwestern and southeastern regions.

The next article in the SI is a review article on the changes and climatic oscillations that occurred in Europe during the Quaternary period and how these changes influenced the diversity of plant habitats and their species composition [11]. This article reviews biogeographical research against the background of data on the distribution of the Hercynian Mountains in connection with data for the Alps, the Carpathians, and the European lowlands. It also discusses the relationships between plant populations in the Hercynian Mountains and other mountain ranges in Europe, as well as the main migration routes of glacial relict species that could have arrived and settled in the Hercynian Mountains during the Ice Age and that survived in isolated habitats.

## 3. Conclusions

The articles included in the Special Issue concern various aspects of current issues in plant taxonomy, systematics, and phylogeography and draw readers’ attention to current knowledge and the latest results of scientific research in this field. Three articles concern the phylogeography of bryophytes, two of ferns, and the remaining articles concern higher plants: orchids, the taxonomy of the genus Aconitum, and species of the genus Cerasus. The last two articles describe biogeographic diversity in Gansu, a region of significant biogeographic diversity located in northwestern China, and an article on changes and climatic oscillations that occurred in Europe during the Quaternary period and how these changes influenced the diversity of plant habitats and their species composition. The articles published in our SI are of great importance for plant taxonomic research, systematics, and phylogeography, and we hope that their recognition and citation will increase.
